# Film Formation Mechanism of Aqueous Polymer Particle Dispersions for Barrier Coating Applications

**DOI:** 10.1021/acsami.5c05234

**Published:** 2025-05-14

**Authors:** Maria Morits, Anneli Lepo, Muhammad Farooq, Monika Österberg

**Affiliations:** † School of Chemical Engineering, Department of Bioproducts and Biosystems, Aalto University, Vuorimiehentie 1, 02150 Espoo, Finland; ‡ R&D and Technology, 86569 Kemira Oyj, P.O. Box 44, 02271 Espoo, Finland

**Keywords:** film formation, barrier coating, polymer, nanoparticles, dispersion coatings, liquid AFM

## Abstract

Dispersion coatings are promising barrier solutions for fiber-based packaging. Among the advantages of water-borne dispersion coatings are easier repulping, recyclability, and composting of fiber packaging compared to other types of coatings. Dispersion coatings can replace fluorochemicals, which pose health hazards in food packaging. The film formation mechanism is the basis for developing barrier dispersion coatings, as a flawless coating structure is a prerequisite for good barrier properties. However, developing cost-efficient dispersion coatings with good film formation properties combined with good barrier and converting properties remains challenging. In this work, we implement atomic force microscopy (AFM) imaging in air and water to study the film formation mechanisms at the nanoscale of a series of styrene–acrylic copolymer barrier dispersion coatings with different surfactant/stabilizer systems and correlate these findings with the barrier properties of the coatings determined by common methods. In particular, AFM is used to characterize the morphology of the films prepared under different conditions, illuminating the effect of both the core polymer chemistry and stabilizing system on film formation. The relationship between the film morphologies and barrier properties of the coatings is subsequently revealed. In addition to AFM, another surface-sensitive technique, quartz crystal microbalance with dissipation monitoring (QCM-D), is used to evaluate the interaction of the dispersion coating particles with cellulosic substrates. The significant impact of the chemical structure of the stabilizer system of the dispersion on the barrier properties of coatings is unraveled by using this approach. Overall, this work reveals the predictive capacity of the AFM technique for evaluating the barrier properties of dispersion coatings.

## Graphical abstract

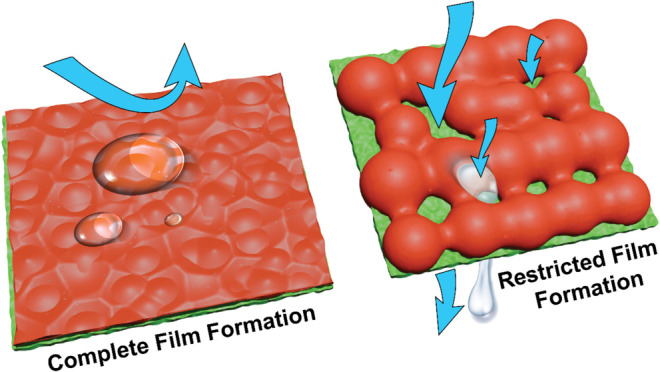

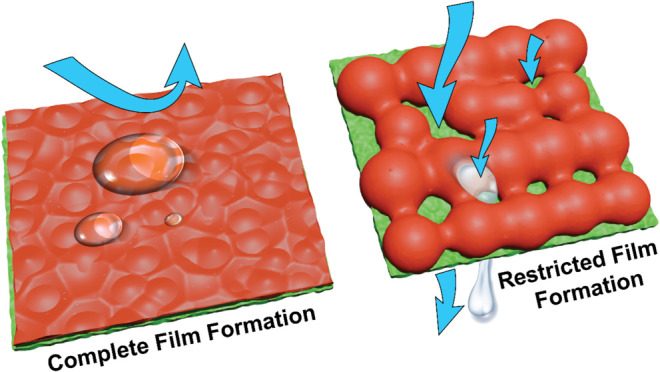

## 1 Introduction

Plastic waste is a big issue for the environment since only a small part of plastic waste is recycled^1,2^ whereas a considerable amount of the rest of the waste ends up in landfills,^3^ litter on land and water bodies,^4,5^ and causes microplastic pollution in the environment as a result of incomplete plastic degradation.^6^ Packaging materials, especially food packaging, comprise a large part of all plastic waste, combined with the short average time the packages are used before being discarded.^7^ Moreover, the production of packaging is growing along with logistics networks and online shopping, as packaging plays a crucial role in food storage, including the packaging of bakeries, instant food, fast food, and take-away food. Increasing the use of plant fiber-based packaging is a way to reduce the use of fossil-sourced plastics and a step toward the more sustainable use of materials. In contrast to the challenging recycling of fossil-based plastic, which is usually mixed and contaminated,^8,9^ paper and paperboard possess much better recycling possibilities.^10^ An additional advantage of plant-based materials is that paper and paperboard fibers that become too short for recycling can be composted. However, paper and paperboard are intrinsically highly porous and hygroscopic materials and, thus, do not possess sufficient barrier properties for food packaging. Paper and paperboard can achieve the required barrier properties through different types of coatings, such as plastic films and dispersion coatings, or by using fluorochemicals.^11^

The use of lamination and polymer extrusion coatings for plant fiber-based materials complicates their recycling. Thus, increasing environmental concerns and tightening regulations regarding packaging and packaging waste^12^ have led to the fact that water-based dispersion coatings have gained recent interest since dispersion coatings allow the reduction of the use of plastic in coated fiber-based packaging and facilitate repulping, recyclability, and composting.^13–16^ Applying dispersions in online coatings is efficient, and dispersions can be applied at high speeds compared to extrusion coatings; thus, dispersion coatings can be cost-efficient for packaging solutions. In terms of the health and safety of workers producing packaging materials, water-based coatings are solvent-free formulations; thus, they have a lower odor and emit fewer volatile organic compounds (VOC), leading to low VOC exposure in the workplace. Finally, dispersion coatings offer safer replacements for fluorochemicals in food packaging.^17^

Despite their advantages concerning environment and safety, it has been challenging to achieve barrier performance similar to thicker laminates or extruded polymer films using dispersion coatings.^18^ The most important task of dispersion coating development is good film formation since the interdiffusion of polymer chains of different particles is lower than in films made from polymer solutions or polymer melts. Poor film formation will lead to pinholes and consequently poor barrier performance.^15^

The barrier performance of dispersion coatings has been the focus of many recent studies, whereas film formation of polymer particles has not received sufficient attention.^19–22^ Earlier studies on the film formation mechanism have mainly focused on typical latex film formation without referencing their performance as coatings. Different theories regarding latex film formation^23^ and experimental methods of latex film formation studies,^24–30^ including atomic force microscopy (AFM),^31,32^ environmental and cryo-scanning electron microscopy, and small-angle X-ray scattering, have been proposed. The methods and results of these studies can be adapted for studies of dispersion coating film formation. However, to date, most studies on the structure of dispersion coatings have focused on the shape and distribution of mineral fillers (pigments) within the coatings and the related improvement of the barrier properties of the coatings.^33–35^ The relationship between the film formation mechanism and barrier properties of polymeric dispersion coatings without mineral fillers has, in contrast, not received adequate attention. Notwithstanding, the possibility of predicting the barrier behavior of dispersion coatings using knowledge of film formation obtained by surface-sensitive techniques would allow the development of coatings that can be applied at higher speeds and are more cost-efficient.

The use of plant-based components for dispersion coatings allows us to further reduce the carbon footprint of the coatings. Starch and its derivatives are widely used in the paper industry as coatings and sizing agents.^36^ Oxidized starch is an excellent stabilizer and can be used for the preparation of dispersion coatings. However, to date, the effect of oxidized starch as a stabilizer on film formation has not been thoroughly studied.

In this work, we investigated the structure–property relationship of barrier dispersion coatings and revealed the effects of the chemical structure and physical properties of surfactants, stabilizers, and core polymers on the film formation of dispersion coatings. Using AFM, we explored the morphology of dispersion particles at the nanoscale both in the wet state and after drying under varying conditions, and identified the key parameters affecting film formation at the nanoscale. The unique ability of AFM to image aqueous dispersions without drying, as well as in ambient air without the need for staining or other sample preparation, enabled us to distinguish distinctly different stages of the film formation process from separate particles to coalescence. The interactions of the dispersions with the cellulosic substrates were further studied using quartz crystal microbalance with dissipation monitoring (QCM-D). These fundamental findings using surface-sensitive techniques were correlated with the barrier properties of the dispersion coatings using standard methods, including the water vapor transmission rate, water absorption test, and oil and grease resistance test.

## 2 Experimental Section

### 2.1 Materials

Poly­(ethylene imine) (PEI, 50 wt %) was purchased from Sigma-Aldrich. The monomers styrene, *n*-butyl acrylate, and acrylic acid (≥99%) were purchased from Acros Organics. The ferrous sulfate heptahydrate catalyst (≥99%) was purchased from Merck. Hydrogen peroxide 30% used as the initiator for polymerization and NaOH 50% used for pH adjustment were purchased from VWR Chemicals. Perfectamyl A4692 starch with a degree of substitution as the average number of carboxylic groups per anhydroglucose unit (0.040–0.048), used as a stabilizer, was purchased from Avebe. Perfectamyl A4692 was oxidized and degraded to a *M*_w_ of 8400 g/mol for the synthesis of an amphiphilic stabilizer. The anionic surfactant Dowfax 2A1 (disodium lauryl phenyl ether disulfonate, *M*_w_ = 574 g/mol) was purchased from EZkem. The materials were used as supplied, except that hydrogen peroxide was diluted with deionized water to 8 wt %. Deionized water was used in all of the polymerizations.

### 2.2 Synthesis of Amphiphilic Stabilizers and Core Polymers of Dispersions

Amphiphilic stabilizers possessing carboxylate charges, consisting of oxidized starch and a hydrophobic polymer, poly­[styrene-*co*-(*n*-butyl acrylate)-*co*-(acrylic acid)], were synthesized. Amphiphilic stabilizers were made by polymerization in the presence of starch and the same monomer mixture as in the subsequent polymerization of the hydrophobic polymer to obtain dispersion; thus, it was assumed that the compositions of the hydrophobic part of the stabilizer and the polymer of dispersion were the same. The polymerization procedure for the synthesis of the core polymers of the dispersion and amphiphilic stabilizers is described below.

The particle dispersions used in this study were prepared by emulsion polymerization. Initiation was done using a hydrogen peroxide–ferrous sulfate redox pair. The amount of hydrogen peroxide was 1.4 wt %, and ferrous sulfate was 0.001 wt % of the monomers. Mixtures of styrene, *n*-butyl acrylate, and acrylic acid in different ratios were emulsified using their corresponding amphiphilic stabilizers, anionic surfactants, or mixtures of anionic surfactants and stabilizers in water. Polymerization was carried out in a 1 L reactor equipped with a cooling/heating jacket, reflux condenser, nitrogen inlet, feeding inlet for the monomer mixture and initiator, and stirrer. Polymerization was performed under a nitrogen atmosphere at 95 °C. The monomer mixture and initiator solution were fed continuously to the reactor base containing water, stabilizer and/or surfactant, and ferrous sulfate heptahydrate for 250 min. The pH of the approximately 50% solids content dispersion was adjusted to 6.2 with NaOH after polymerization. The dispersions obtained were used for the coating preparation. The compositions and molecular weights of the obtained copolymers, poly­[styrene-*co*-(*n*-butyl acrylate)-*co*-(acrylic acid)], are presented in Table S1 in Supporting Information.

### 2.3 Preparation of Coatings

Coatings were prepared by applying the dispersions on A4 paperboard sheets by using a laboratory bar coater (K Control Coater, RK Print Coat Instruments) at a speed of 10 and a rod number of 3. The coatings were dried under an IR dryer (InfraRR) for 60 s. All sheets were conditioned under standard climate conditions at 23 ± 1 °C and 50% ± 2% relative humidity for 4 h before and after the coating and weighed to determine the applied coat weight by the weight difference. The coat weight applied to the paperboard was approximately 15 g/m^2^. The paperboard substrate had a basis weight of 216 g/m^2^. The Bendtsen air permeability of the paperboard, measured from the coated side, was 296 mL/min, and the Cobb_300_ value was 45 g/m^2^. The basis weight was measured according to ISO 536, Bendtsen air permeability according to ISO 5636-5, and Cobb_300_ according to the ISO 535 standard.

Prior to testing, all paperboard sheets were conditioned under standard climate conditions at 23 ± 1 °C and 50% ± 2% relative humidity for a minimum of 4 h.

### 2.4 Grease Resistance: KIT Test

Grease resistance was evaluated by using the Tappi T559 standard method. Twelve test solutions, composed of varying ratios of castor oil, toluene, and *n*-heptane, were used to evaluate the samples’ resistance to a certain solution based on visual observations. The kit test was run for flat-noncreased and nonfolded surfaces, creased surfaces, and folded surfaces. Creasing was done using a Cyklos GPM-450. The sample was folded along the creasing lines by using a Cobb roller with a mass of 10 kg. One drop of the test solution was pipetted onto the sample; the solution was left on the sample for 15 s, whereupon the solution was wiped off the sample. The result was observed visually as the absorption of the solution. Two parallel measurements were performed, and the results are given as the representative full value.

### 2.5 Water Absorption: Cobb Test

The Cobb test was performed in accordance with the ISO 535 standard. Water absorption was determined as the amount of water absorbed in 300 s. A sample was cut into a piece of 150 × 150 mm, which was larger in diameter than a cylinder corresponding to a test area of approximately 100 cm^2^. The sample was weighed and placed on a metal base plate with the test surface facing upward. The cylinder was placed on top of the sample and locked using a holder. 100 mL of water was poured into the cylinder. Water was poured out 15 s before the end of the test duration. Subsequently, the sample was carefully positioned between the absorbent paperboard sheets. A Cobb roller was applied once in each direction across the sample. After removal, the sample was weighed. The difference in the weight before and after water absorption was calculated and expressed in g/m^2^. Two parallel measurements were performed, and the results are given as the representative full value.

### 2.6 Water Vapor Transmission Rate (WVTR)

The water vapor transmission rate (WVTR) of the sheets was measured with a Systech Illinois AquaSense Model 7101 Water Vapor Permeation Analyzer, the corresponding software being Systech Illinois 7101. This method conforms to ASTM F-1249, ISO 15105-2:2003, ISO 15106-3:2003, and DIN 53122-2 standards. The WVTR measurement was performed at 23 °C and 50% RH using the MASK option with an extended range, a bypass time of 15 min, and a purge level of 10. The results of this measurement are expressed as g/(m^2^·day). Two parallel measurements were performed, and the results are given as the representative full value.

### 2.7 Ethanol Stain Test

A 60 mm × 60 mm piece was cut from the coated sheet. A creasing machine was used to crease the sample in the cross direction and the machine direction. The sample was folded along the creasing lines using a Cobb roller with a mass of 10 kg to ensure that the folding force was the same each time. Creasing was done using a Cyklos GPM-450. A saturated ethanol solution of methyl red was prepared. Six drops of the ethanol solution were dripped onto the sample surface. The solution was spread and dried using a kitchen towel. Digital photographs were captured of both the tested and reverse sides of the sample for visual evaluation of the coated surface.

### 2.8 Olive Oil Test

A 60 mm × 60 mm specimen was cut from the coated sheet and placed onto a glass plate, followed by the placement of two cotton patches in the middle of the sample surface. A total of 6 drops of olive oil were dripped on the cotton patches, and a 50 g weight was placed on the top of the patches. The test setup was placed in a preheated 40 °C oven for 2 h. The samples were scanned as soon as possible after taking them out of the oven to observe any oil stains.

### 2.9 Differential Scanning Calorimetry (DSC)

A Mettler Toledo DSC 3+ instrument was used to determine the *T*_g_ of the dispersion particles. Measurements were conducted in the temperature interval from −65 to 100 °C at heating rates: the first heating run was 20 °C min^–1^, followed by cooling, and the second heating was 5 °C min^–1^ under a 50 mL min^–1^ nitrogen flow. Before analysis, the samples were freeze-dried. A standard aluminum sample holder with a capacity of 40 μL was used. The glass transition temperatures were determined based on the second heating thermograms.

### 2.10 Minimum Film Forming Temperature (MFFT)

The MFFTs of the dispersion particles were determined according to the ISO 2115 standard. A Rhopoint Instruments MFFT 90 equipped with a temperature gradient plate was used for the measurements. The plate was smeared with glycerine and covered with a foil. A few lines of dispersion were applied on the covered gradient plate with an applicator with a 45 μm gap. Each measurement was conducted for 2 h.

### 2.11 Particle Size Analysis

The size distributions of the dispersion particles were measured using a Malvern Zetasizer Nano ZS90 (Malvern Instruments Ltd., United Kingdom). The size distribution was analyzed using the Zetasizer software supplied with the device.

### 2.12 Atomic Force Microscopy (AFM)

A Bruker MultiMode 8 Atomic Force Microscope was used for AFM measurements. Measurements were conducted in the tapping mode in air and water. NCHV-A probes were used for AFM in air, and SCANASYST-FLUID+ probes were used for AFM in water. Research NanoScope Analysis 1.5 software (Bruker) was used to process the images. The only image correction applied was the flattening of the height images.

### 2.13 Sample Preparation for AFM Measurements

Two sets of samples were prepared for AFM measurements. Both sets of samples were deposited on silica wafers coated with positively charged PEI. Before the adsorption of PEI, the silica surfaces were cleaned and treated in an Ozonator for 20 min. 0.25 wt % aq. PEI solution was adsorbed onto the silica wafers for 15 min, after which the silica wafers were rinsed with pure deionized water and thoroughly dried with nitrogen gas.

For the preparation of the first set of samples for AFM in air, dispersions of 20 wt % were poured on top of PEI-coated surfaces and quickly dried using nitrogen gas to form a multilayer film. The silica wafers were attached to special magnetic disks for the AFM samples using double-sided tape.

For preparing never-dried samples for AFM in water, PEI-coated silica surfaces were immersed in dispersions of 0.05 wt % and kept in the fridge for 12 h before AFM imaging, allowing the particles to adsorb to the positively charged surface. The substrates were then rinsed with deionized water and kept wet until measurements by adding a few water droplets on the coated substrate.

Free-standing films were prepared by casting the dispersions on the plastic surfaces at room temperature and 90 °C. For the AFM measurements of free-standing films, small pieces (approximately 1 cm^2^) were cut off from the free-standing films and attached to the surface of the special magnetic discs using double-sided tape.

For the AFM measurement of coatings, similar to the preparation of the samples of free-standing films, small pieces (approximately 1 cm^2^) were cut off from the coated paperboards and attached to the magnetic discs using tape.

### 2.14 Quartz Crystal Microbalance with Dissipation Monitoring (QCM-D)

For the adsorption of the synthetic polymer dispersion, QCM-D sensors coated with cellulose nanofibers (CNF) were placed in the flow module, and deionized (DI) water or buffer (pH 8) was injected at a 100 μL/min flow rate. After baseline stabilization, polymer dispersions of 1.0 g L^–1^ at their native pH or pH 8 were pumped into the measurement cells. The frequencies (*f*_3_, *f*_5_) and dissipation factors (*D*_3_, *D*_5_) of the third and fifth overtones were continuously monitored until a plateau region was obtained. DI water or buffer was reinjected to remove any loosely bound polymer particles from the CNF-coated substrates. The obtained QCM-D micrographs were analyzed for adsorption behavior.

### 2.15 Sample Preparation for QCM-D Measurements

To evaluate the film formation, the adsorption of synthetic polymer dispersions onto the cellulosic surfaces was performed. For this purpose, thin films of cellulose nanofibers were deposited onto QCM-D gold sensors to serve as a model of a pure cellulose substrate. The CNF thin films on the QCM-D gold sensor were prepared as follows: The sensors were first cleaned by exposure to UV/ozone for 15 min, followed by rinsing with Milli-Q water and nitrogen drying. Then, the sensors were coated with PEI as the anchoring polymer. After 10 min, PEI was rinsed off the crystal and gently dried with N_2_, followed by the adsorption of the CNF dispersion and then spin coating at 4000 rpm for 1 min. The CNF spin-coated sensors were rinsed with DI water and dried gently with nitrogen gas.

## 3 Results and Discussion

### 3.1 Structure and Properties of Dispersion Particles

This study aimed to investigate how stabilizer characteristics and polymer chemical composition influence the film formation process in dispersion coatings intended for use as grease and water barriers on paperboard. The film formation and morphology data were correlated with standard barrier performance tests, as depicted in Scheme 1.

**Scheme 1:**
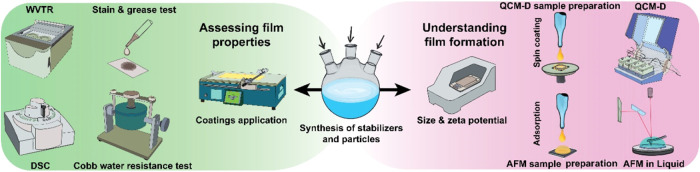
Overview of Techniques for Fundamental Understanding and Application Testing of Dispersion Coatings

Three different polymer compositions, stabilized by three distinct systems, were examined. The samples were designated according to the stabilizer type (amphiphilic, anionic, or a combination of the two) and their glass transition temperatures (*T*_g_) (approximately 10, 30, or 50 °C). Table 1 summarizes the key dispersion properties of the samples, and Table S1 shows the chemical composition of the core copolymers.

**Table 1:** Compositions and Characteristic Properties of the Dispersions

		stabilizer wt %	surfactant wt %	*Z*_Ave_^a^ nm		
dispersion sample	surfactant/stabilizer	of the hydrophobic polymer			*T*_g_ °C	MFFT °C
Amphi10	amphiphilic stabilizer	29.1	0	151	9	–3
Amphi30		29.1	0	118	30	25
Amphi50		29.1	0	97	53	53
Mix10	amphiphilic stabilizer and anionic surfactant	17.5	2	102	8	1
Mix30		17.5	2	88	31	26
Mix50		17.5	2	103	54	60
Anion10	anionic surfactant	0	2.3	184	10	3
Anion30		0	2.3	188	33	26
Anion50		0	2.3	176	53	60

a The *z*-average size was determined by DLS.

### 3.2 Morphology of the Particles in Aqueous Media

To examine the morphology of polymer particles with different stabilizers, we conducted AFM imaging of polymer particles in water with *T*_g_ and minimum film formation temperatures (MFFT) below room temperature (Figure 1). AFM analysis revealed that all particles were spheroidal. Measurements based on NanoScope Analysis of two 25 μm^2^ images for each sample showed that Amphi10 had a mean height of 58 nm and a diameter of 210 nm, Mix10 measured 97 nm in height and 168 nm in diameter, and Anion10 measured 105 nm in height and 217 nm in diameter. Notably, large particle aggregates were not observed in any of the samples.

**Figure 1:**
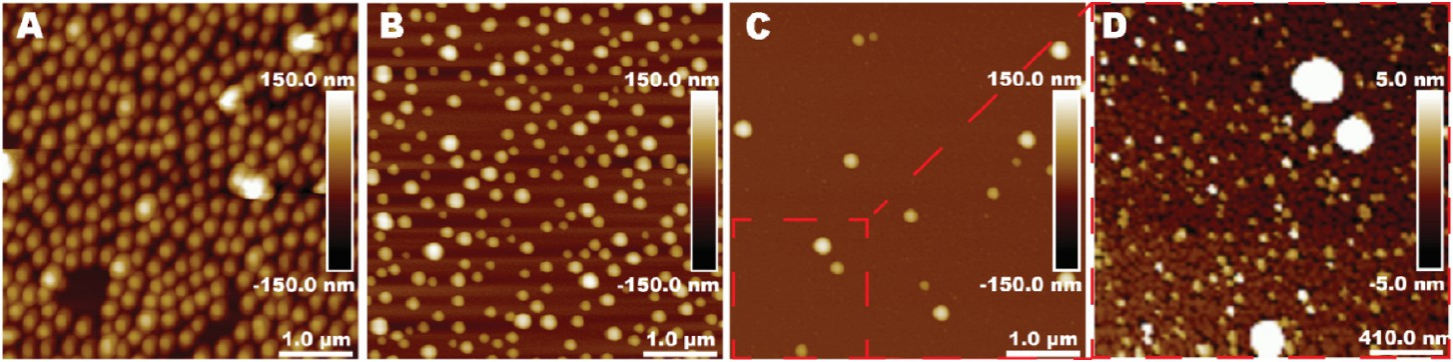
AFM images of dispersion particles imaged in the tapping mode in water. Topography (height) images of (A) Amphi10, (B) Mix10, and (C) Anion10, and (D) magnified area of the image (C) marked with a red square with a height scale from −5 to 5 nm.

The lower mean height of the Amphi10 particles compared to that obtained by a zeta sizer can be caused by the good adsorption of the Amphi10 particles to the PEI-coated silica wafers and partial deformation of the soft particles on the surface. The large difference between the height and diameter of the Amphi10 particles supports this conclusion. For hard, nondeformable particles, the height would be the most accurate size obtained by AFM since the diameter is exaggerated due to tip convolution.^37^

Particles stabilized with the amphiphilic stabilizer (Amphi10) exhibited higher surface coverage on the positively charged surfaces compared to particles stabilized with either a mixture of stabilizer and surfactant or surfactant alone. The surface coverage of Anion10 particles in water is surprisingly low, as shown in Figure 1C. Figure 1D shows the presence of small 5–7 nm particles, which we speculate are micelles formed from free surfactant molecules. Similar particles were not observed in the other samples, even at that height scale. We hypothesize that there was an excess of surfactant in the sample Anion10 and, during the preparation of the sample, small negatively charged surfactant micelles were formed. We suggest that the presence of micelles substantially decreased the surface coverage of Anion10 particles because the micelles competed with the polymer particles for attachment to the positively charged surface. Additionally, negatively charged particles of different sizes repel each other, thus preventing high surface coverage. The particles stabilized with the surfactant had a higher charge density than those particles stabilized with the amphiphilic stabilizer.

### 3.3 Effect of Stabilizer and Kinetics of Drying on the Fusion of the Nanoparticles

Understanding the film formation mechanism is crucial for developing effective dispersion barrier coatings for paperboard and other substrates. This process is typically divided into four distinct stages, as illustrated in Figure 2 ^38^ Stage I (wet stage): the dispersion is in its initial liquid state, with particles suspended in the aqueous phase. Stage II (particle packing): the particles form a close-packed array with water-filled voids remaining between them. Stage III (particle deformation): the particles undergo deformation and become densely packed, driven by the need to reduce surface energy and by capillary forces while still maintaining their individual identities. Stage IV (particle coalescence): in the final stage, polymer chains from adjacent particles begin to interdiffuse, causing the boundaries between the particles to blur and ultimately form a continuous film.

**Figure 2:**
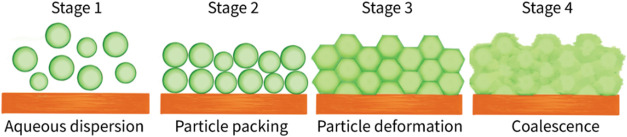
Film formation stages of the polymer particles.

The AFM analysis yields intriguing results, as illustrated in Figure 3, which displays AFM topographic images of the N_2_ gas-dried thin films prepared from all of the dispersions at room temperature and the corresponding schematics of the film formation stages of the dispersions with *T*_g_ 10 °C. Although the *T*_g_ of Amphi10 was well below the room temperature, the film formation in this sample stopped at stage III. The particles were tightly packed and deformed, forming dodecahedral structures. However, the particles did not fuse into a continuous film (Figure 3A and B). In contrast to Amphi10, no particles were visible in the height image of Anion10 after drying (Figure 3I and J). This indicates that the absence of an amphiphilic stabilizer allows for the final stage (IV) film formation. On the other hand, the dispersion stabilized by the mixture of the stabilizer and surfactant (Mix10, Figure 3E and F) illustrates a transitional phase between stage III and stage IV of the film formation mechanism. In this state, some individual particles remain visible, while the majority are significantly deformed, indicating partial progression toward a continuous film. The thin film of dispersion Mix10 exhibited a morphology that was intermediate between that of Amphi10 and Anion10, resembling a network of connected particles and indicating incomplete film formation. With an increase of *T*_g_, the effect of the stabilizer becomes less pronounced since the higher *T*_g_ of the polymer hinders film formation at room temperature (Figure 3C,D,G,H,K,L). While no tendency for fusion was observed for the polymer particles with *T*_g_ 30 or 50 °C stabilized with the amphiphilic stabilizer, the particles of the Anion30 dispersion (Figure 3H) looked softer around the edges than the other particles of dispersions with *T*_g_ above room temperature, which can be the effect of the MFFT of Anion30 being lower than its *T*_g_ and the absence of amphiphilic stabilizer. The comparison of all samples (Figure 3) revealed two factors that negatively influencing the film formation: the increase in the amount of amphiphilic stabilizer and the growth of core polymer *T*_g_. The negative effect of the amphiphilic stabilizer can be easily seen in the comparison of the samples with *T*_g_ below the room temperature (Figure 3A,D,G).

**Figure 3:**
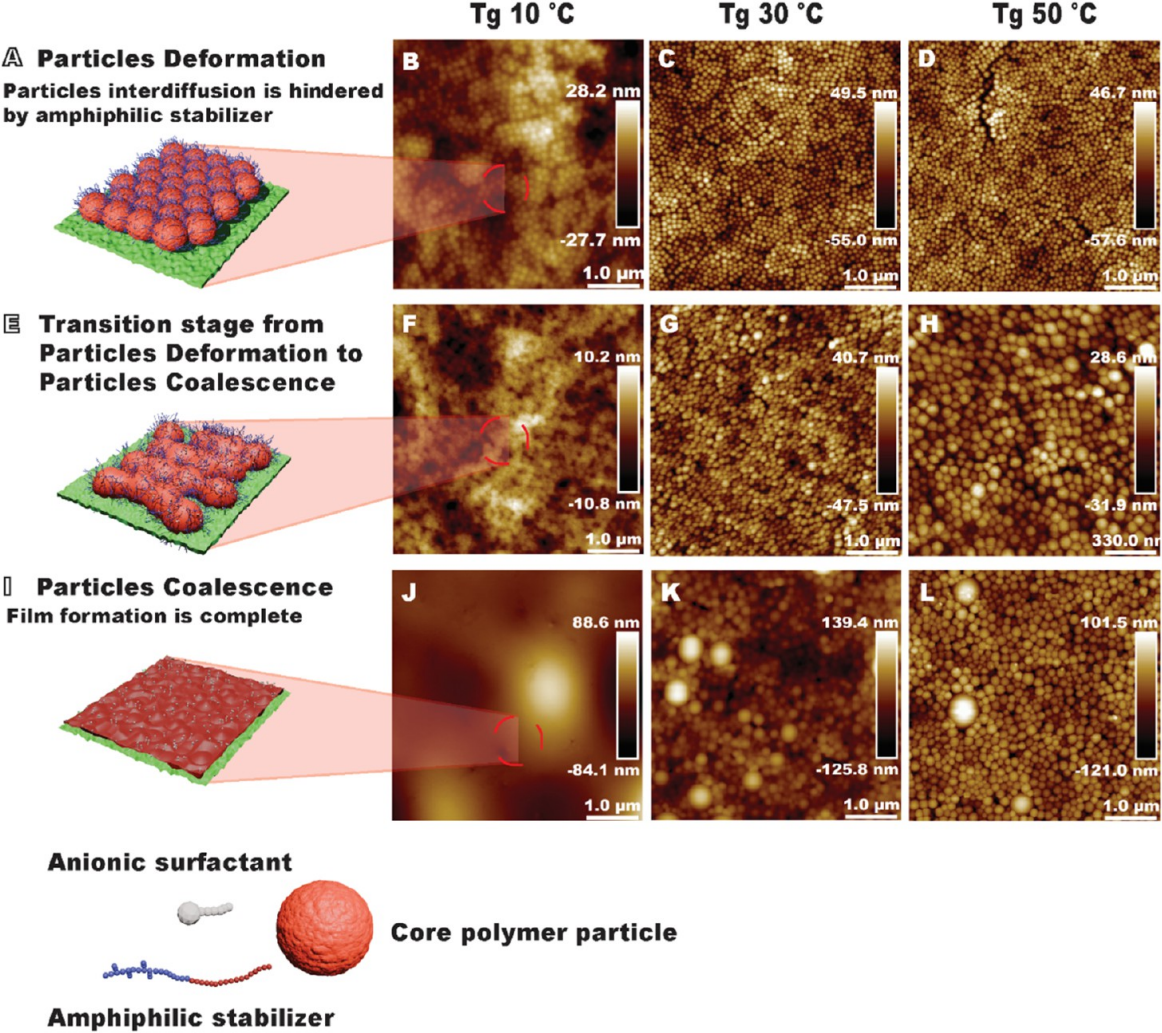
Effect of stabilizer and core polymer composition on the morphology of dried dispersion coatings. The left column shows schematic illustrations of (A) Amphi10, (E) Mix10, and (I) Anion10 and the right column shows topography (height) AFM images of (B) Amphi10, (C) Amphi30, (D) Amphi50, (F) Mix10, (G) Mix30, (H) Mix50, (J) Anion10, (K) Anion30, and (L) measured in ambient air.

While the effect of *T*_g_ was expected, the effect of the amphiphilic stabilizer was surprising. We hypothesize that the oxidized starch chains in the amphiphilic stabilizer are the reason for hampering the film formation. Starch molecules are bulky and interact with each other through hydrogen bonds. These properties decrease the mobility of the amphiphilic stabilizer. The system containing starch also had a higher molecular weight than the others. However, the effect of *T*_g_ for film formation was more decisive than the difference in *M*_w_. Thus, the film formation of the dispersions stabilized by the amphiphilic stabilizer requires more time and possibly more energy (Figure 3B) than that of the dispersions stabilized by only an anionic surfactant (Figure 3J).

To verify the hypothesis that the amphiphilic stabilizer significantly slows down film formation, the effect of sample drying time or water evaporation rate on film formation was revealed for the sample Amphi10 with *T*_g_ below room temperature stabilized with amphiphilic stabilizer (Figure 4). It was observed that quick drying of the dispersion (less than 1 min) preserved the morphology of the nanoparticles (Figure 4A). In contrast, a longer time of drying led to more deformation of the particles (Figure 4B), followed by outflow of the core polymer (Figure 4C). This can be explained by the higher mobility of stabilizer molecules in water during drying. This is in line with previous observations of classic latex films that dealt with continuous films at stage IV and explained the lower quality of the quickly dried film with non-optimum conformation of latex’s polymer chains.^28^ However, since the nanoscale morphology of dispersion coatings has not received much attention, we could not find previous studies showing the effect of the stabilizer on film formation.

**Figure 4:**
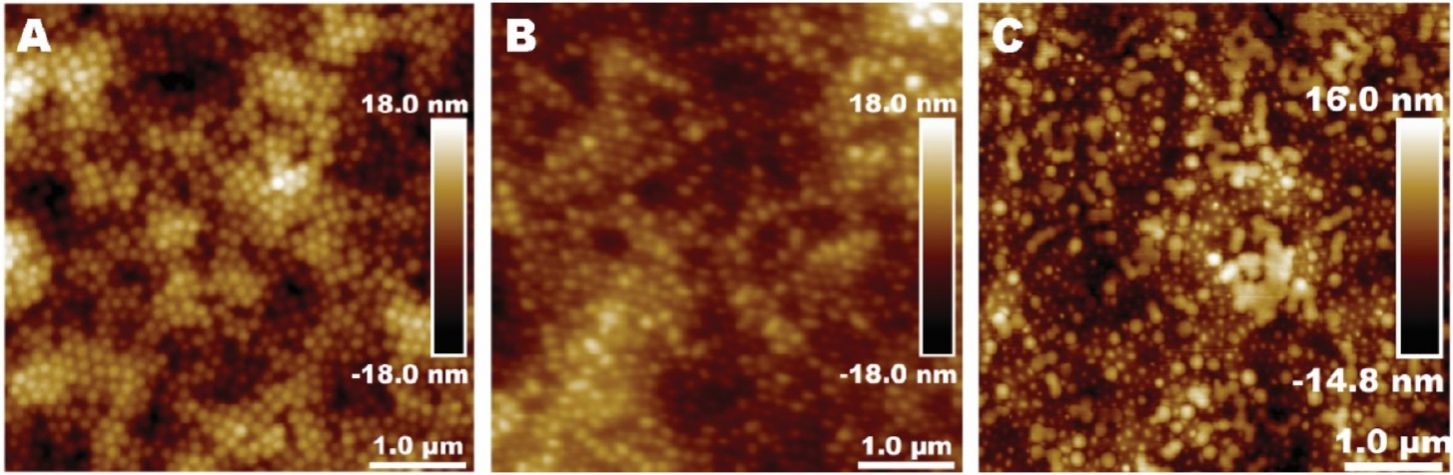
Effect of the sample drying time on film formation of the sample Amphi10. (A) Fast drying with N_2_ gas (less than 1 min) on a silica wafer, (B) slow drying (3 h) on a silica wafer, and (C) slow drying (more than 3 h) of a free-standing film.

### 3.4 Morphology of Free-Standing Films Made from the Dispersions

Free-standing films with thicknesses of 1–2 mm were prepared to eliminate the influence of the substrate on film formation. Figure 5 shows free-standing films cast at room temperature and at 90 °C. The morphology of the film samples prepared at room temperature mainly depended on the *T*_g_ of the particles (Figure 5A–C) since the drying time of the free-standing films far exceeded that of the thin films described above (several hours vs tens of seconds). The negative effect of the stabilizer on the formation of free-standing films was less pronounced. The sample Amphi10 with a *T*_g_ below room temperature demonstrates the particle coalescence (Figure 5A), which corresponds to the final stage of film formation. The image of sample Amphi30 with *T*_g_ about 30 °C demonstrates deformation of the particles with partial coalescence (Figure 5B), and finally, the sample Amphi50, with the highest *T*_g_ in the series, demonstrated particles at the packing stage—II stage of the film formation according to the literature^38^ (Figure 5C). Amphi50 was the most brittle sample of the series because of its incomplete film formation. Since the particles of Amphi50 were solid at room temperature, they could not form a densely packed structure, leading to a small contact area between the particles and consequently weak adhesion between the particles. Preparation of the films at 90 °C led to softening of the particles’ core and coalescence of the particles in all samples (Figure 5D–F). Films made from the dispersions were stabilized by both the mixture of the amphiphilic stabilizer and surfactant, and only the surfactant followed the same trend as observed for the samples stabilized by the amphiphilic stabilizer (Figures S1 and S2).

**Figure 5:**
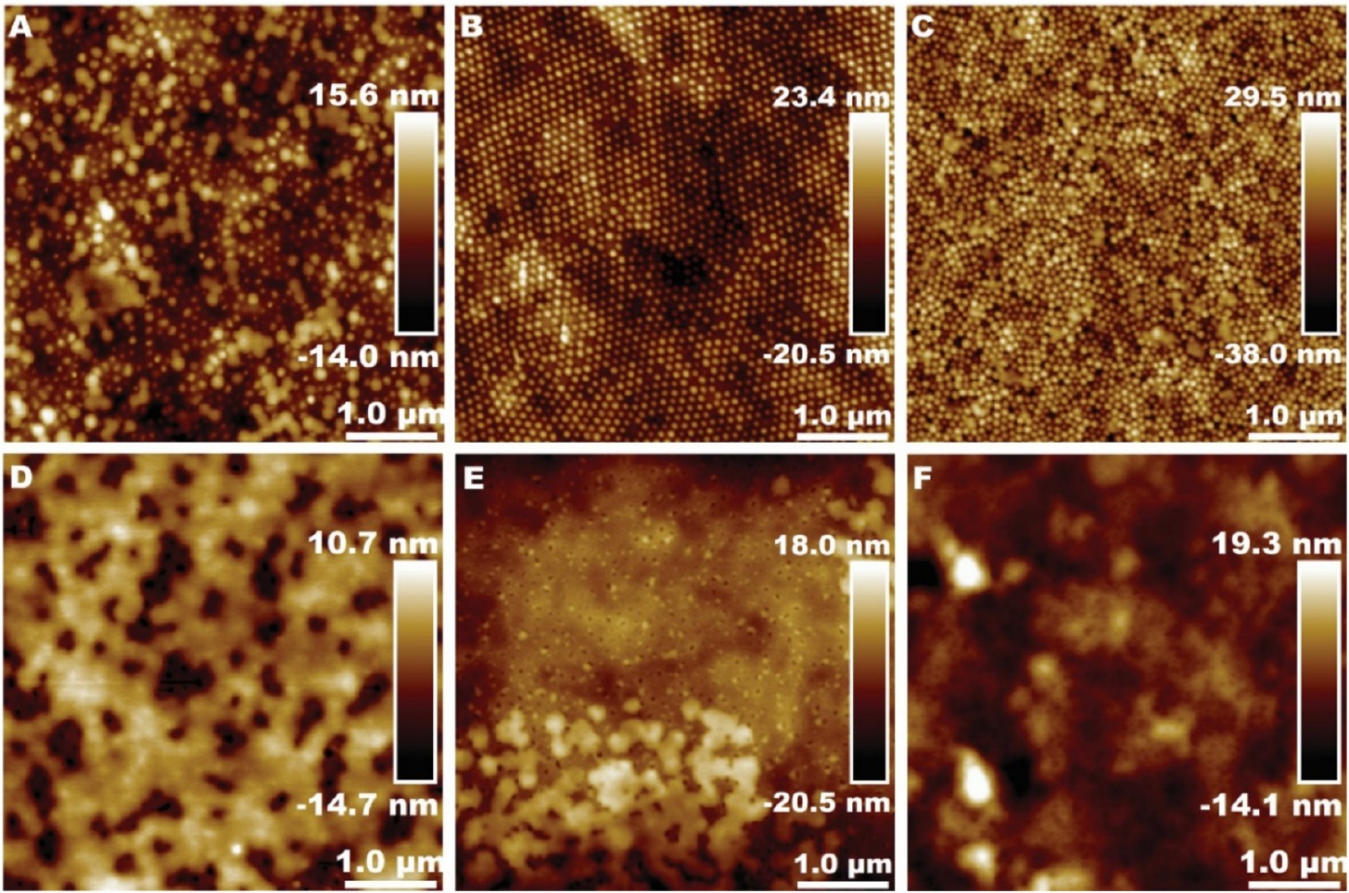
Morphology of free-standing films made of polymer particles stabilized by amphiphilic stabilizers with different *T*_g_ values and dried at different temperatures. (A) Amphi10, (B) Amphi30, and (C) Amphi50 were dried at room temperature (top row); and (D) Amphi10, (E) Amphi30, and (F) Amphi50 were dried at 90 °C. The AFM images were scanned in the tapping mode in air at room temperature.

### 3.5 Barrier Properties of the Dispersion Coatings

Barrier properties of the coatings are crucial for their applications in food packaging and largely determine their area of use. In this work, the board was coated with dispersion samples to assess the barrier properties of the dispersion coatings and correlate the film formation results with their performance as barrier coatings.

The film formation properties of dispersion coatings severely impact their barrier properties since film formation largely determines the structure of the coating film. In particular, slow kinetics of film formation can lead to the formation of a weaker film due to the low interdiffusion of polymer chains of neighboring particles or non-optimal polymer conformation. Moreover, slow kinetics can lead to flaws. Defects in coatings are detrimental to their barrier performance. One of the important factors affecting film formation and barrier properties is the *T*_g_ of the dispersion polymer.^32,36^ On the one hand, a lower *T*_g_ is preferred in terms of film formation and to obtain continuous and ductile films. However, too high polymer chain mobility can decrease the gas barrier properties of the coatings.^39^

An ethanol staining test was conducted to visualize the discontinuities of the coated surfaces, such as pinholes and voids (Figure 6). The ethanol staining test is a screening experiment designed for the straightforward identification of poor-quality coatings. The samples were creased and folded to evaluate the coating film’s mechanical resistance against cracking. Dispersions with a *T*_g_ of around 10 °C had only a few stained small spots at the creasing and folding lines, which can be attributed to the folding resistance of the coatings. The stain was able to pass through the creasing lines of an Amphi10-coated sheet (Figure 6A). We suggest that the amphiphilic stabilizer hinders the film formation during fast drying, leaving more brittle polysaccharide-rich areas. Mix10 and Anion10 also had small stained spots on the flat coated surfaces of the paperboard substrate. In contrast, the stain did not pass through the coated paperboards to the reverse sides of the sheets coated with Mix10 and Anion10 (Figure 6D and G), correlating with the better film formation observed using AFM (Figure 3). Coatings with *T*_g_ 30°C dispersions demonstrated penetration of the stain solution through the coatings along the creasing and folding lines due to severe cracking at these areas, regardless of the stabilizer composition. The reason behind these coatings’ brittleness is the glass transition temperature above the test temperature and the low elasticity of the polymer coatings.^40,41^ These ethanol staining tests were applied to identify the coatings that were inappropriate for barrier applications.

**Figure 6:**
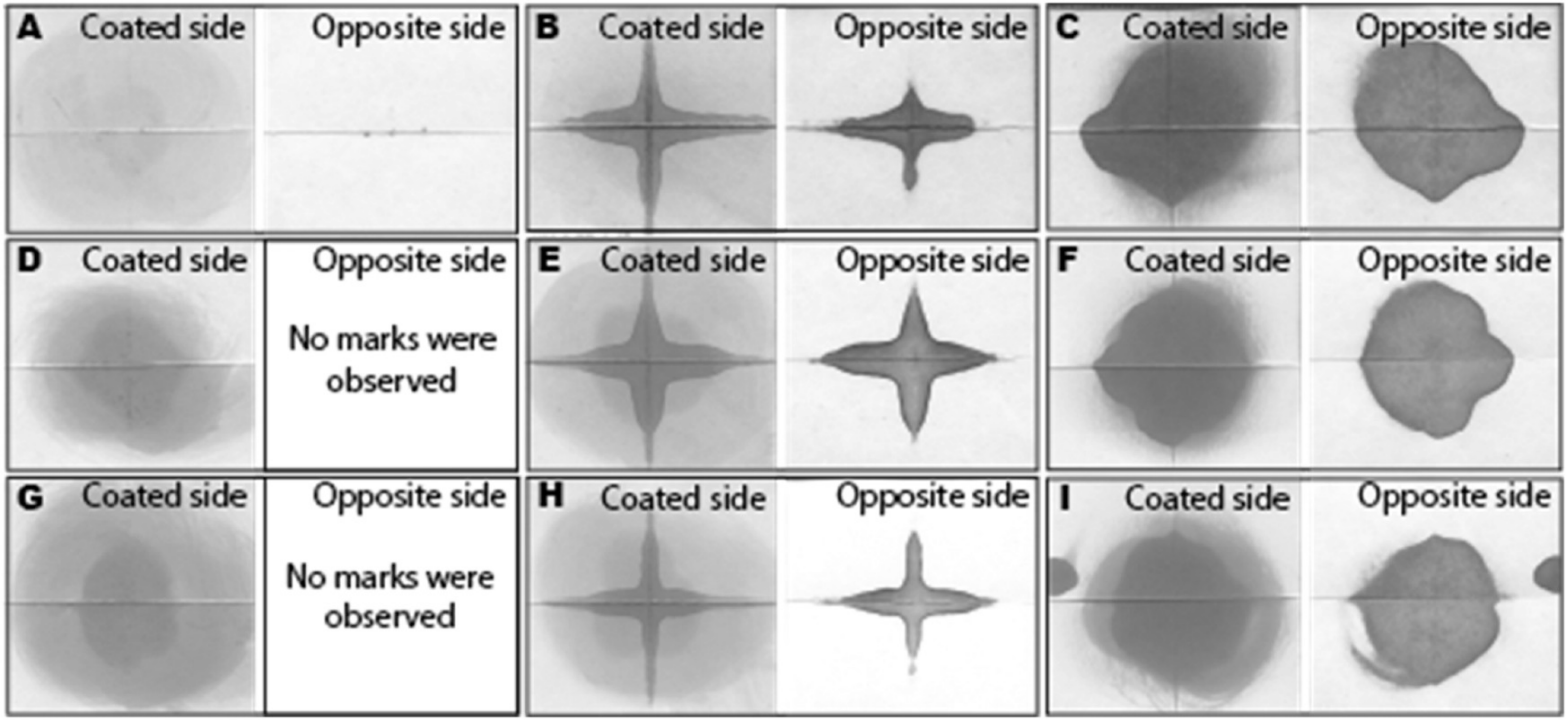
Ethanol stain test of dispersion coatings applied to a paperboard substrate. Photographs of stained coated surfaces, and uncoated opposite sides of the tested sheet corresponding to (A) Amphi10, (B) Amphi30, (C) Amphi50, (D) Mix10, (E) Mix30, (F) Mix50, (G) Anion10, (H) Anion30, and (I) Anion50 dispersions.

The surface morphology of the dispersion coatings applied to the paperboard substrate is presented in Figure 7. The fact that the ethanol stain solution passed through the samples with a *T*_g_ of 50 °C is explained by cracks in the coatings observed by optical microscopy (Figures 7A,E,I, S5). The cracks led to significant deterioration of all barrier properties of the samples coated with the dispersions having a *T*_g_ of 50 °C, as discussed further (Figure 8). The cracks were present in all *T*_g_ 50 °C samples and were not affected by the type of stabilizer.

**Figure 7:**
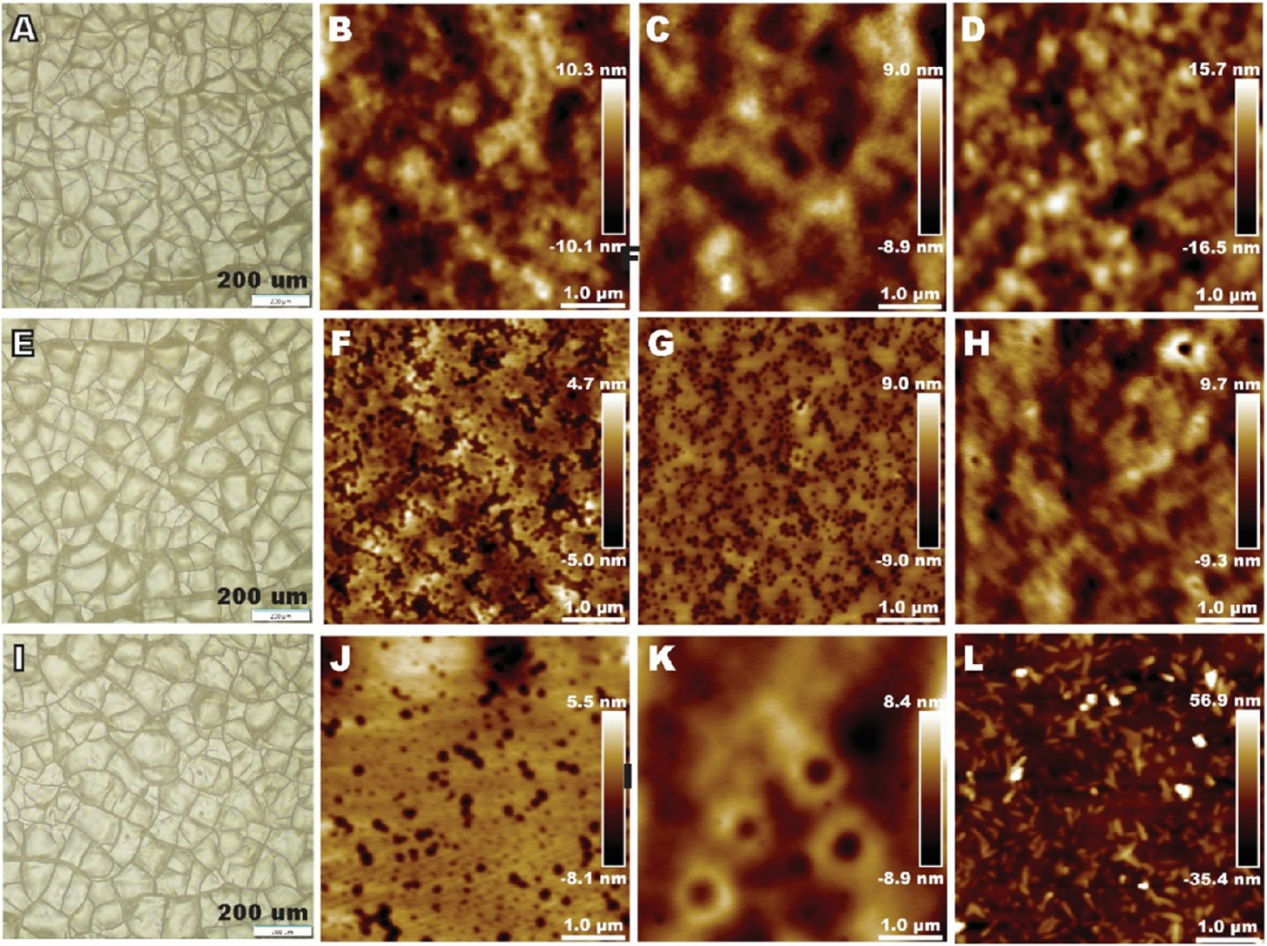
Surface morphology of the dispersion coatings applied to a paperboard substrate. Optical microscopy images of (A) Amphi50, (E) Mix50, and (I) Anion50. AFM height images of the dispersion coatings of (B) Amphi10, (C) Amphi30, (D) Amphi50, (F) Mix10, (G) Mix30, (H) Mix50, (J) Anion10, (K) Anion30, and (L) Anion50.

**Figure 8:**
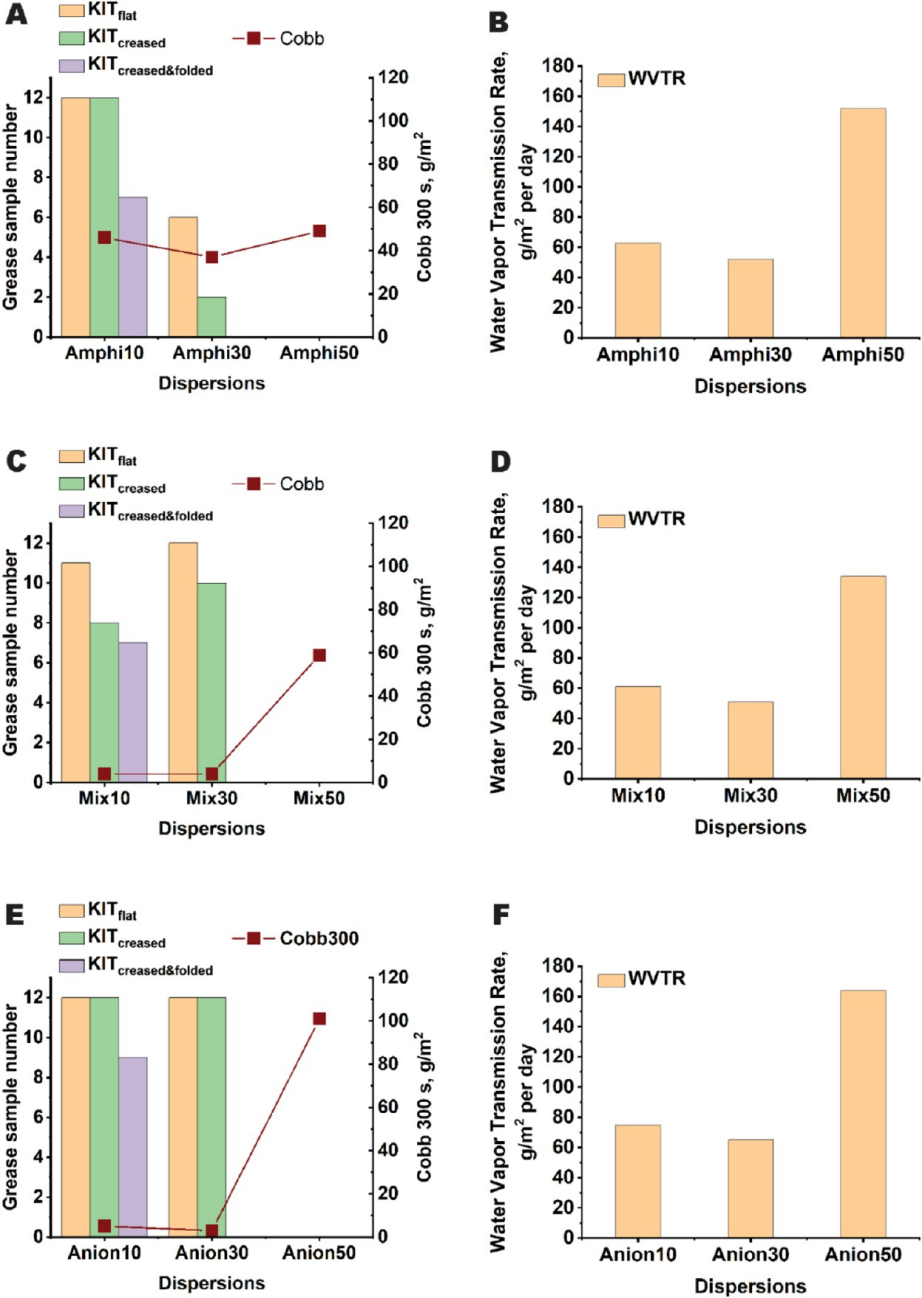
Grease, water, and WVTR tests of the dispersion coatings applied to a paperboard substrate. (A) KIT and Cobb_300_ values and (B) WVTR of dispersion coatings stabilized by the amphiphilic stabilizer, (C) KIT and Cobb_300_ values, and (D) WVTR of dispersion coatings stabilized by a mixture of amphiphilic stabilizer and surfactant, and (E) KIT and Cobb_300_ values and (F) WVTR of the dispersion stabilized by the anionic surfactant. The KIT, Cobb, and WVTR results are presented as representative full values of the two measurements.

Dispersion coatings undergo lateral mechanical stress during film formation and drying.^42–44^ One reason for the crack formation is the low deformability of the samples with high *T*_g_ and thus insufficient stress dissipation, which led to “mud cracking”—the appearance of microcracks on a film surface resembling cracks of dried muddy sediment—of the coatings at the final stages of drying.^26^ Moreover, the short heating—cooling cycle did not allow for an optimal conformation of polymer chains, leading to additional mechanical stress in the material.

Overall, according to AFM, the particles of all dispersion coatings fused under the used coating preparation conditions (Figure 7). However, nano-pits were visible in the AFM images of all samples, except the sample Anion50 (Figure 7L). The nano-pits observed likely resulted from insufficient polymer chain mobility during rapid thermal processing (60 s at 120 °C). This accelerated drying created a competition between solvent evaporation and polymer chain relaxation/diffusion processes, restricting polymer interdiffusion at particle interfaces, as evaporation occurs too quickly for optimal particle rearrangement and coalescence. The addition of suitable plasticizers may promote particle mobility, leading to better film formation. Alternatively, the formation of nano-pits can be due to phase separation caused by the presence of a stabilizer or surfactant.

For a more detailed assessment of the barrier properties of the dispersion coatings, the KIT test, Cobb_300_ test, olive oil test, and WVTR measurements were performed. Figure 8 shows the barrier properties of the coatings applied on the base paperboard. To replace fluorochemicals, dispersion coatings should be oil and grease resistant. In this work, the KIT test was used to determine the grease resistance of the coatings (Figure 8A,C,E). The KIT_flat_ test for all samples with *T*_g_ of 10 or 30 °C, except Amphi30, demonstrated high values of 11 or 12, corresponding to excellent grease resistance. The grease resistance of the coating of all samples with a *T*_g_ of around 10 or 30 °C was further confirmed by the olive oil test, where Amphi10 and Amphi30 had equal resistance compared to Mix10, Mix30, Anion10, and Anion30 (Figure S4). The poorer result of Amphi30 can be a consequence of the weaker resistance of the amphiphilic stabilizer to the organic solvents toluene and heptane used in the KIT test. In addition to the effect of the stabilizer solubility on grease permeability,^36^ porosity affects the grease resistance of coatings, and thus, in the case of sample Amphi30, the negative effect of the amphiphilic stabilizer on film formation became more pronounced since the drying of coatings was very fast. However, no cracks were observed with an optical microscope (Figure S3B). In contrast to the samples with a *T*_g_ of 50 °C, the higher polymer chain mobility of the samples with a lower *T*_g_ provided better quality and mechanical properties of the formed coatings.

The KIT test results for the creased and creased-folded samples provide information on the mechanical resistance of the coatings. Creased and folded samples undergo larger deformation than only creased samples, which is reflected in the better KIT_creased_ values in comparison with KIT_creased&folded_. The results of the ethanol stain test were confirmed by the poor KIT_creased&folded_ results of the samples with T_g_ of around 30 °C. In contrast, Amphi10, Mix10, and Anion10 demonstrated high KIT_creased_ and KIT_creased&folded_ values, indicating good elasticity and durability of these coatings (Figure 8A,C,E). Regarding the effect of chemical structure on the coating properties, the benzene ring in styrene monomers imparts rigidity and restricts mobility in the styrene-*n*-butyl acrylate copolymer chains. Increasing the styrene to *n*-butyl acrylate ratio leads to higher *T*_g_ values and enhances both stiffness and ultimate mechanical stress of the copolymer film.^45^ As styrene contributes significantly to polymer stiffness, the effects of stabilizers became more pronounced in the *T*_g_ 10 °C samples compared to the *T*_g_ 30 °C samples, which contain more styrene. Conversely, the higher proportion of *n*-butyl acrylate in the *T*_g_ 10 °C coatings improved flexibility and film formation, providing moderate tolerance for creasing and folding.

The presence of an amphiphilic stabilizer combined with rapid drying slowed down the interdiffusion of polymer chains of neighboring particles, as observed in the AFM images (Figure 3B). This may have left more brittle starch-rich areas in the coatings, leading to poorer KIT_creased_, KIT_creased&folded_, and ethanol stain results for the starch-containing Amphi and Mix samples compared to Anion samples having similar *T*_g_ values. It should be noted that the structure and mechanical properties of the substrate also affected the KIT test results. Hence, the results from different reports should be compared with caution. However, since the same base paperboard was used for all samples in this study, the comparison between samples done here is valid.

The water resistance of the dispersion coatings is presented in Figure 8A,C,E. In this work, the coating’s resistance to water was tested for 300 s (Cobb_300_). It should be noted that if the coating has poor water resistance, the water adsorption properties of the substrate paperboard will substantially contribute to the Cobb values. This was also observed in this study. Compositions, including a higher amount of hydrophobic styrene–acrylic copolymers, Anion10 and Anion30, provided low Cobb_300_ results and demonstrated good water resistance. However, the coatings made of Mix10 and Mix30 dispersions demonstrated the best water resistance despite containing the amphiphilic stabilizer, which included a hydrophilic polysaccharide block (Figure 8C). The samples Amphi10 and Amphi30 demonstrated the worst Cobb_300_ values among the samples with *T*_g_ of 10 and 30 °C. The reason behind the observed poor properties may be the presence of a large amount of amphiphilic stabilizer (about 30 wt % of particles) containing hydrophilic polysaccharide blocks. As observed by AFM, the amphiphilic stabilizer hindered particle interdiffusion and the formation of a continuous polymer film (Figure 3B); thus, we can assume that the polysaccharide block acts as an additional channel for water transition through the coating. Polysaccharides could even swell, which would facilitate the movement of water. In Mix10 and Mix30, the amount of the amphiphilic stabilizer was smaller, enabling the formation of a more consistent film quality during drying. Dispersion coatings with a *T*_g_ of 50 °C showed visible cracking and the poorest water vapor barrier performance, with WVTR values comparable to those of the uncoated base sheet (161 g/m^2^ per day) (Figure 8B,D,F). Between the *T*_g_ 10 and 30 °C samples, stabilizer composition had minimal influence on WVTR under standard climate conditions, though Amphi and Mix dispersions performed slightly better than Anion dispersions. This may be attributed to denser packing in polysaccharide-containing compositions, which is notable since high hydrophilic polysaccharide content would typically worsen performance, as demonstrated in the Cobb test results for Amphi-coated sheets.

Dispersions with *T*_g_ of 10 and 30 °C formed coatings of better quality and thus possessed better water vapor barrier properties in the range of 40–80 g/m^2^ per day, which is only slightly inferior to the data presented in other publications on dispersion coatings.^46^ AFM images of coatings with *T*_g_ of 10 and 30 °C demonstrate the presence of nanosized pits. Thus, one can assume that the presence of nanopores in the coatings may have affected their gas barrier properties. Additionally, the samples stabilized only by the anionic surfactant contained a somewhat higher percentage of acrylic acid in the core polymer to obtain good stability of the dispersions without the presence of the amphiphilic stabilizer (Table S1). This could have led to the deterioration of the water vapor barrier properties in comparison to other coatings with *T*_g_ of 10 and 30 °C.^47^ However, since the base paperboard also affects the measured WVTR, comparison with other results is not straightforward.

### 3.6 Interaction of the Dispersions with Cellulosic Substrate

In addition to the properties of the stabilizer and core polymer, the interaction between the dispersions and substrate is critical for ensuring optimal film formation and performance. The dispersions studied here are intended for application to cellulosic substrates, where they form thin films that act as barriers. To evaluate these interactions, the *in situ* adsorption of the dispersions onto cellulosic substrates was measured using QCM-D. These measurements offer insights into how well the dispersions adhere to cellulosic surfaces, which is essential for achieving the desired film properties. Thin films of mechanically disintegrated cellulose nanofibrils were used to simulate the pulp fiber substrate.

The capability of QCM-D for real-time monitoring of the adsorption process was utilized to study the adsorption kinetics of dispersion on the cellulosic substrate (gold sensor covered with CNF). Figure 9 shows the adsorption of dispersions with a *T*_g_ of 50 °C onto a cellulosic substrate. The adsorption results for the other dispersions are presented in Figure S5. An instant decrease in the oscillation frequency (Δ*f*_3_) (Figure 9A) and a simultaneous increase in the dissipation (Δ*D*_3_) (Figure 9B) were observed as the dispersions reached the CNF surfaces, suggesting rapid initial adsorption. The main driving force for adsorption is expected to be an increase in entropy due to the release of water molecules from around the dispersion particles and from the CNF film. Surprisingly, Anion50 demonstrated higher adsorption, observed as a larger decrease in frequency Δ*f*_3_compared to the less charged Amphi50 and Mix50, despite its higher negative charge caused by the presence of the surfactant on the surface of the particles. These results can be explained by the fact that water coupled to the adsorbed material also affects the observed frequency changes. The corresponding dissipation curves are in agreement with this assumption (Figure 9B). The Δ*D*_3_ for Anion50 was larger than the dissipations of Amphi50 and Mix50, which can correspond to a looser and swollen viscoelastic layer of the adsorbed material. It is expected that particles repelling each other would not form a dense, rigid layer. Following this logic, Mix50, containing an anionic surfactant, demonstrated higher changes in Δ*f*_3_ and Δ*D*_3_ than the less charged Amphi50. For a more accurate comparison of the viscoelastic properties of the adsorbed colloidal films, we plotted the change in dissipation versus the change in frequency for the dispersions with a *T*_g_ of 50 °C (Figure 9C). Since the adsorbed mass is proportional to the change in the QCM-D resonant frequency, such a plot allows the comparison of the dissipation values at the same adsorbed masses visually. As shown in Figure 9C, the change in dissipation of the Anion50 dispersion was higher than the change in dissipation of the Amphi50 dispersion at similar Δ*f*_3_ values, and it began to surpass the dissipation of Mix50 when the resonant frequency change reached about −3 Hz. This supports the assumption that the Anion50 dispersion formed a looser viscoelastic layer of adsorbed material compared with Mix50 and Amphi50 dispersions on the surface of the QCM-D crystal.

**Figure 9:**
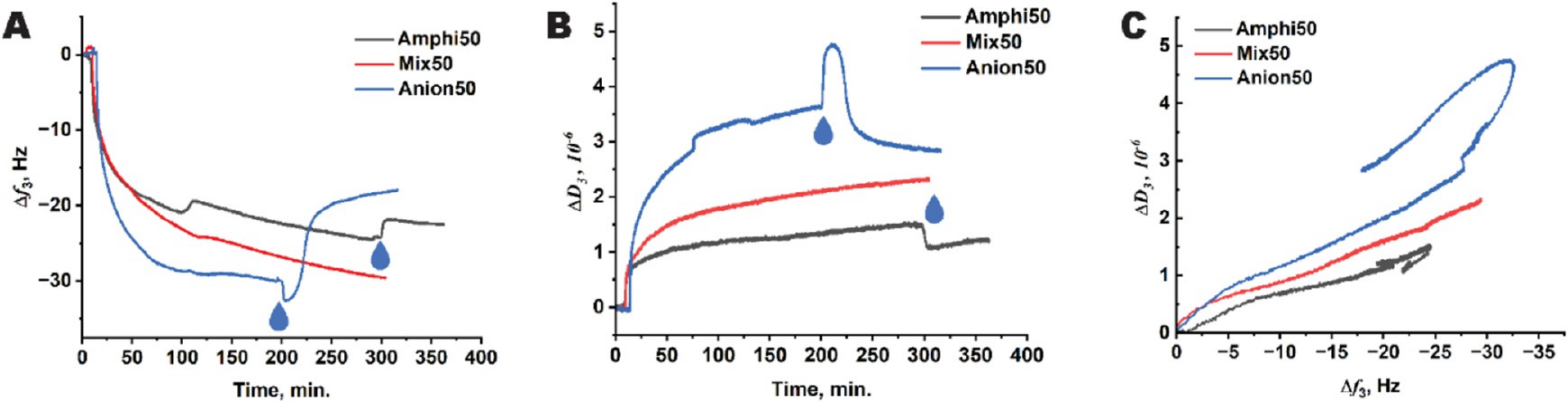
Direct adsorption of the *T*_g_ 50 °C dispersions with different stabilizer systems on cellulose was monitored using QCM-D. (A) Changes in the third overtone of resonant frequency Δ*f*_3_ upon adsorption of the dispersions Amphi50, Mix50, and Anion50 onto the cellulosic model film. (B) Changes in the third overtone dissipation Δ*D*_3_. The points of injection of pure water are marked with droplet signs. (C) Changes in the third overtone dissipation Δ*D*_3_ versus the third overtone of the resonant frequency. The points of injection of pure water are marked with droplet signs.

Additionally, significant desorption of Anion50 particles from the cellulosic substrate due to rinsing of the QCM-D crystal with DI water (Figure 9A) demonstrated the poor attraction of the particles to the substrate. There was a temporary drop in the frequency and a temporary jump in the sample’s dissipation at the moment of injection of DI water (Figure 9), which can be explained by the additional loosening of the adsorbed layer before the particles’ desorption. The desorption of the Amphi50 sample upon rinsing is significantly smaller, as seen by a smaller increase in the frequency.

## 4 Conclusions

This work revealed a strong correlation among the film formation mechanisms, nanoscaled film morphology, and the corresponding barrier properties of dispersion coatings. We further gained insight into the effects of the polymer *T*_g_, the chemistry of the stabilizer systems, and coating conditions on the film formation process. Revealing the structures of the coatings at the nanoscale allowed us to understand how the amphiphilic stabilizer containing oxidized starch hindered the film formation of the dispersion coatings, significantly affecting their barrier properties. Overall, nanoscale investigations provide predictive capabilities for more rational and efficient barrier coating design. AFM has proven to be a viable tool for such studies. More specifically, the nanoarchitecture information obtained through AFM imaging can clarify which aspects of the system are decisive for film formation and thus guide development in the right direction. In the future, we plan to extend this set of techniques to enhance the development of biobased barrier dispersion coatings.

## Supplementary Material



## Notes

Main characteristics of polymers used for the preparation of dispersions (Table S1); AFM images of the morphology of films made of the dispersions stabilized by the mixture of the amphiphilic stabilizer and anionic surfactant (Figure S1); films made of the dispersions stabilized by the anionic surfactant (Figure S2); surface morphology images of the dispersion coatings with *T*_g_ of 10 and 30 °C applied to paperboard obtained by optical microscopy (Figure S3); photographs of olive oil resistance test of the dispersion coatings applied to the paperboard (Figure S4); QCM-D kinetics of adsorption of the dispersions with *T*_g_ of 10 and 30 °C onto the model cellulose film (Figure S5) (PDF)
