# Supplementary material for: Film Formation Mechanism of Aqueous Polymer Particle Dispersions for Barrier Coating Applications
Source: ACS Appl Mater Interfaces. 2025 May 14;17(25):37068–80. doi: 10.1021/acsami.5c05234 (PMC12203457; doi:10.1021/acsami.5c05234)
Supplement: Supplementary file 1 [file am5c05234_si_001.pdf]

# Supporting Information

# Film formation mechanism of aqueous polymer particle dispersions for barrier coating applications

*Maria Morits [a], Anneli Lepo [b], Muhammad Farooq [a], Monika Österberg* [a].*

[a]Aalto University, School of Chemical Engineering, Department of Bioproducts and Biosystems, Vuorimiehentie 1, 02150 Espoo, Finland

[b] R&D and Technology, Kemira Oyj, P.O. Box 44, 02271 Espoo, Finland.

* Corresponding author: monika.osterberg@aalto.fi, phone +358505497218

KEYWORDS: film formation, barrier coating, polymer, nanoparticles, dispersion coatings, liquid AFM

**Table S1.** The main characteristics of polymers used for the preparation of the dispersions

| Sample | Composition | | | Polymer characteristics |
|---|---|---|---|---|
| | n-Butyl acrylate (wt.%) | Styrene (wt.%) | Acrylic acid (wt.%) | $M_w$ (Da) |
| Amphi10 | 58 | 41 | 1 | 486300 |
| Amphi30 | 45 | 54 | 1 | 362500 |
| Amphi50 | 32 | 67 | 1 | 348300 |
| Mix10 | 58 | 41 | 1 | 234950 |
| Mix30 | 45 | 54 | 1 | 119900 |
| Mix50 | 31 | 65 | 4 | 121300 |
| Anion10 | 57 | 39 | 4 | 147200 |
| Anion30 | 43 | 53 | 4 | 180000 |
| Anion50 | 31 | 65 | 4 | 139900 |

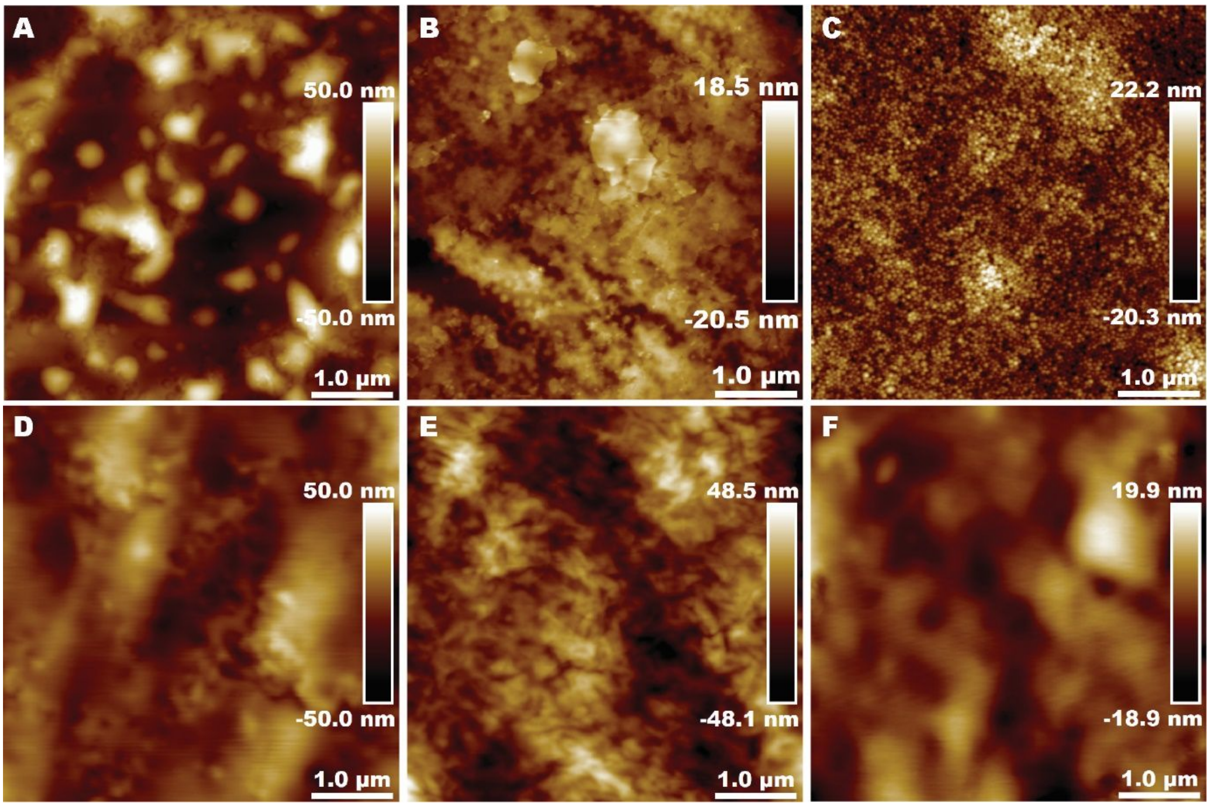


**Figure S1.** Morphology of the films made of particles stabilized by the mixture of the amphiphilic stabilizer and anionic surfactant. A) Mix10, B) Mix30, and C) Mix50 dried at room temperature (top row), and D) Mix10, E) Mix30, and F) Mix50 dried at 90 ˚C. AFM measurements were performed in tapping mode in the air at room temperature.

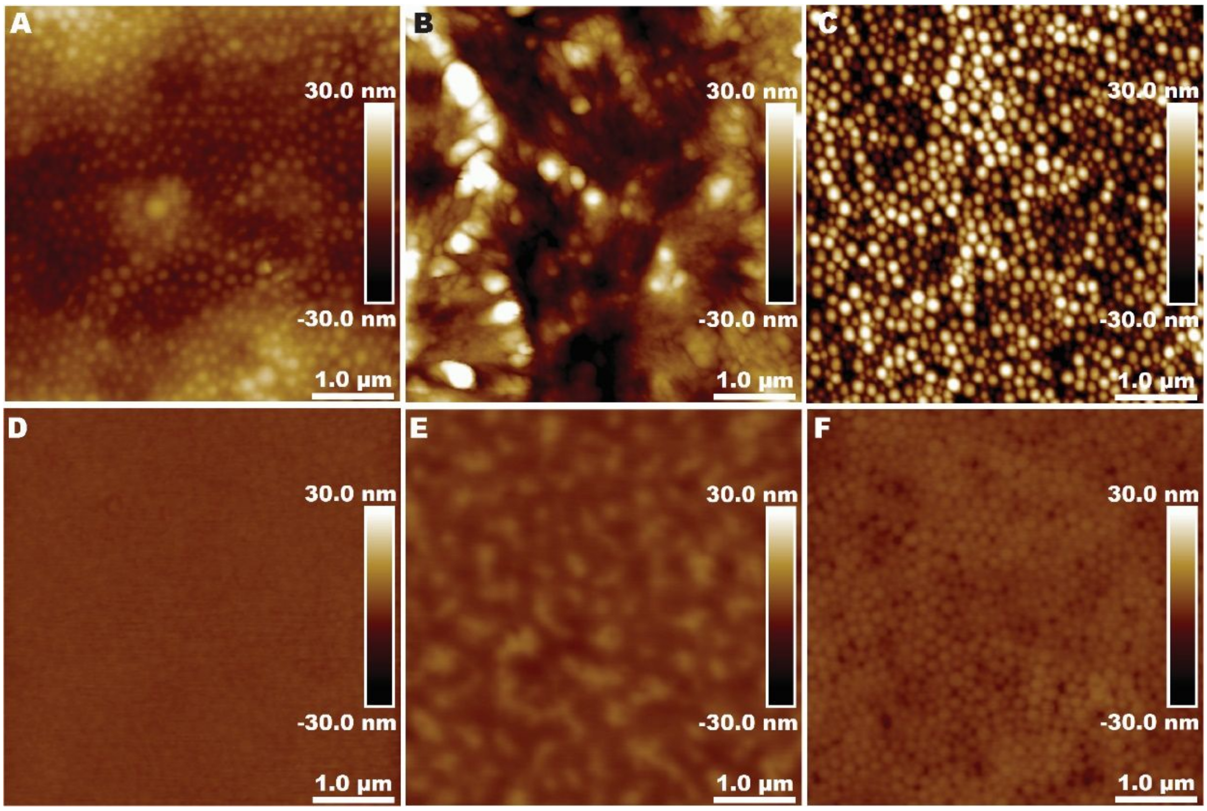


**Figure S2.** Morphology of the films made of dispersion particles with different Tg stabilized by anionic surfactant. A) Anion10, B) Anion30, and C) Anion50 dried at room temperature (top row), and D) Anion10, E) Anion30, and F) Anion50 dried at 90 ˚C. AFM measurements were done in tapping mode in air at room temperature.

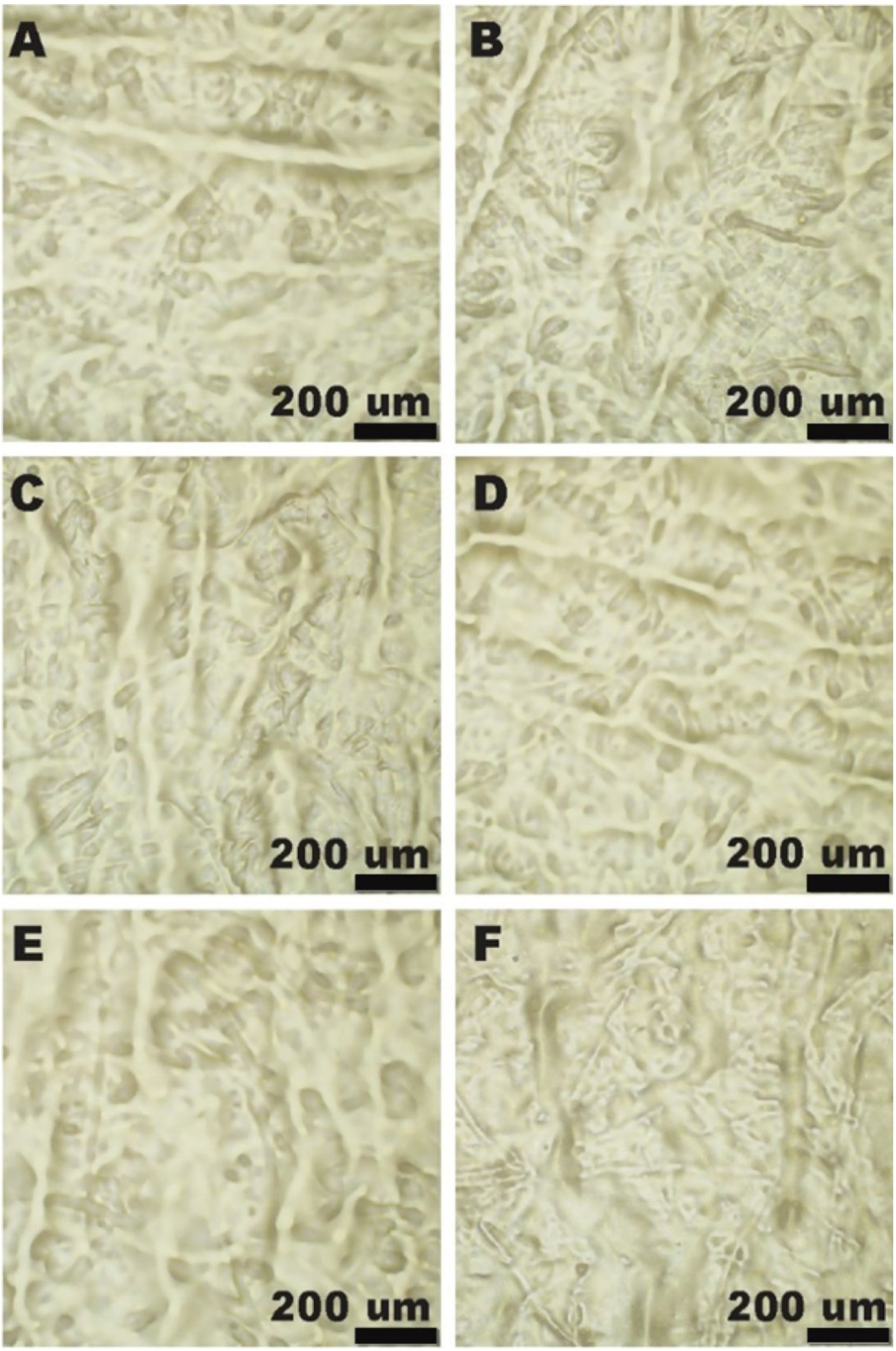


**Figure S3.** Surface morphology of the dispersion coatings applied on paper board imaged using optical microscope. A) Amphi10, B) Amphi30, C) Mix10, D) Mix30, E) Anion10, and F) Anion30. The coatings were dried under an IR dryer (InfraRR) for 60 seconds. Thereafter,

sheets were conditioned at the standard climate conditions at 23 ˚C ± 1 ˚C and 50 % ± 2 % relative humidity for at least 4 hours.

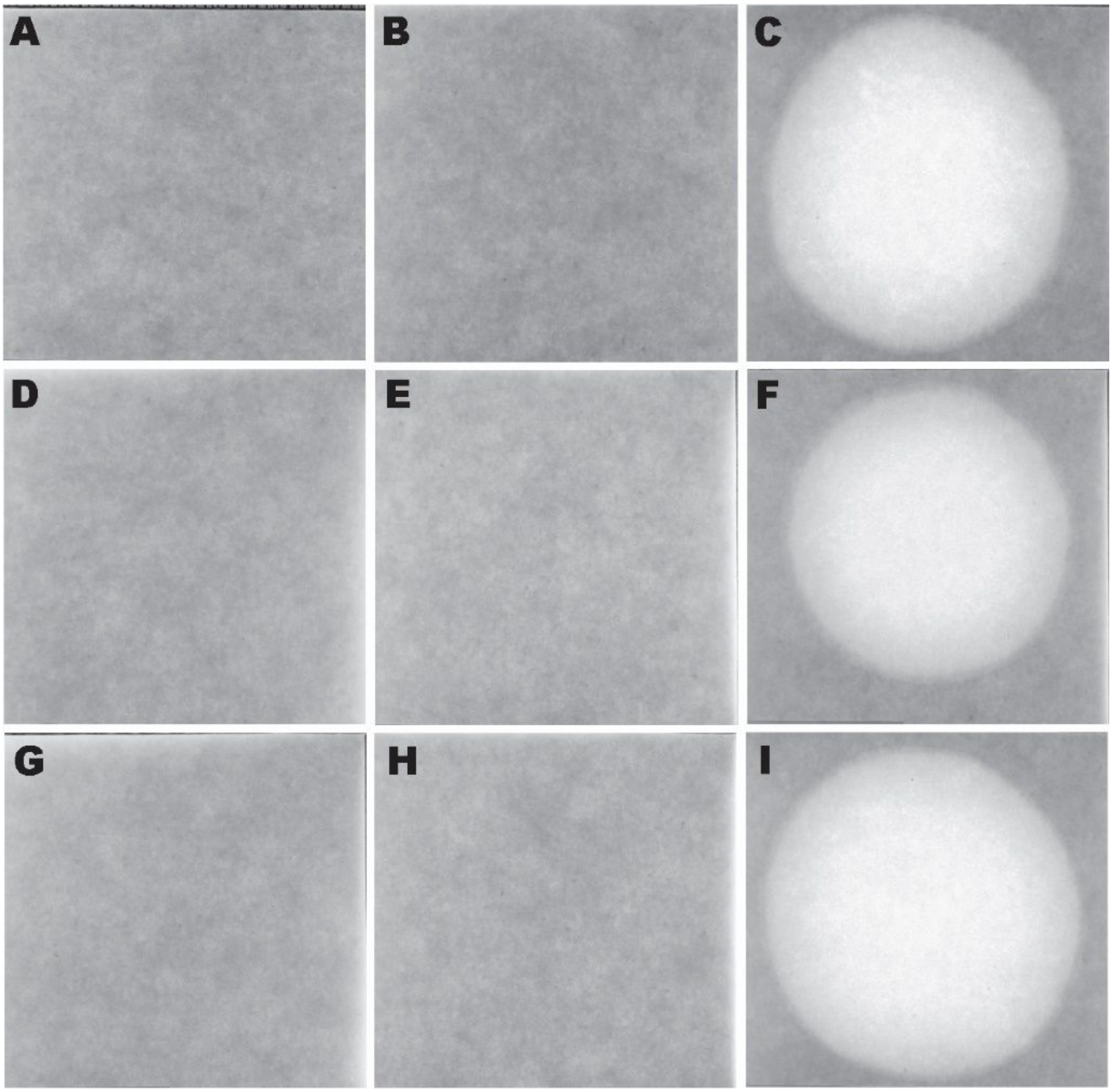


**Figure S4.** Olive oil resistance test of the dispersion coatings applied on paper board. A) Amphi10, B) Amphi30, C) Amphi50, D) Mix10, E) Mix30, F) Mix50, G) Anion10, H) Anion30, and I) Anion50.

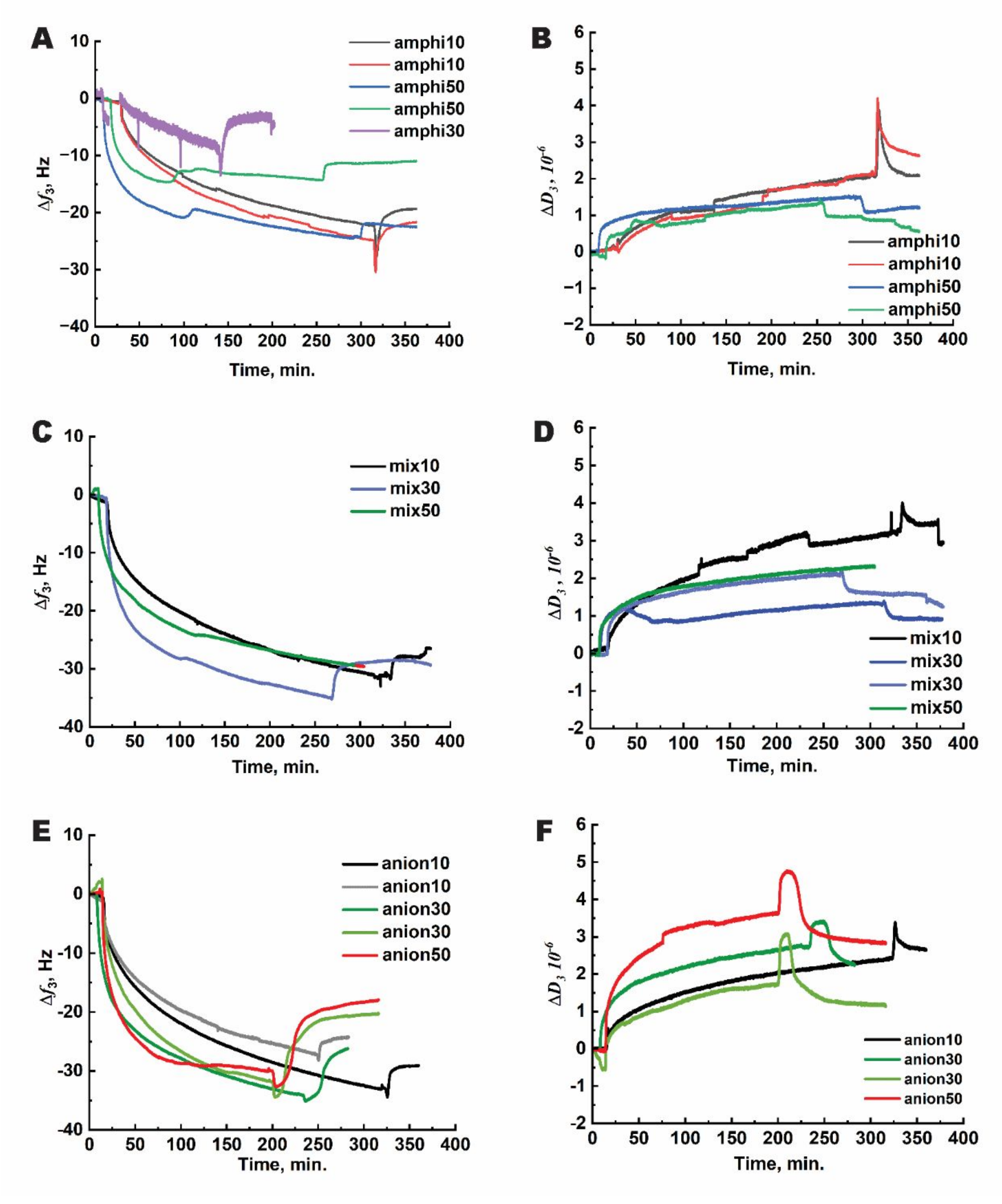


**Figure S5.** In situ QCM-D detection of adsorption of the dispersion onto the model CNF film coated onto the QCM-D sensor surface. Changes in the third overtone of resonant frequency $\Delta f_3$ due to adsorption of the dispersions stabilized with A) amphiphilic stabilizer, C) mixture of amphiphilic stabilizer and anionic surfactant, and E) anionic surfactant. Changes in the third overtone dissipation ΔD due to adsorption of the dispersions stabilized with B) amphiphilic

stabilizer, D) mixture of amphiphilic stabilizer and anionic surfactant, and F) anionic surfactant.
